# Trends and determinants of hospitalization costs for umbilical hernia: a 13-year retrospective analysis from 2012 to 2024

**DOI:** 10.3389/fsurg.2026.1748565

**Published:** 2026-04-09

**Authors:** Baoshan Wang, Qiuyue Ma, Xiaoli Liu

**Affiliations:** 1 Department of Hernia and Abdominal Wall Surgery, Beijing Chaoyang Hospital, Capital Medical University, Beijing, China; 2 Department of Evidence-Based Medicine Center, Beijing Chaoyang Hospital, Capital Medical University, Beijing, China

**Keywords:** cost determinants, health economics, hospitalization costs, surgery, umbilical hernia

## Abstract

**Background:**

Umbilical hernia is a common surgical condition that imposes significant economic burdens on healthcare systems. Despite its prevalence, factors influencing hospitalization costs associated with umbilical hernia repairs remain inadequately understood, particularly in China. This study aimed to analyze trends in hospitalization costs and identify key determinants influencing these costs over a 13-year period.

**Methods:**

We retrospectively analyzed data from 1,578 adult patients who underwent umbilical hernia repair surgery at Beijing Chaoyang Hospital from January 2012 to December 2024, including 146 emergent/urgent cases and 1,432 elective cases. Patient demographic information, clinical characteristics, surgical methods, and cost components were collected from the hospital's electronic medical record system. Univariate and multivariate linear regression analyses were conducted to identify independent predictors of total hospitalization costs, which were log-transformed to approximate normality.

**Results:**

A total of 1,578 patients were analyzed, with a mean hospitalization cost of 32,218.17 ± 18,624.60 CNY. Hospitalization costs showed an upward trend from 21,756.86 CNY in 2012, peaking at 41,314.14 CNY in 2021, followed by a decrease to 25,692.89 CNY in 2024. Material costs consistently constituted the largest proportion of total hospitalization expenses. Multivariable linear regression analysis identified several independent factors associated with higher total hospitalization costs, including later admission years (2016–2019: +30.4%, *P* < 0.001; 2020–2024: +45.4%, *P* < 0.001), laparoscopic repair (+107.9%, *P* < 0.001), mesh use (+108.2%, *P* < 0.001), ICU admission (+68.6%, *P* < 0.001), general anesthesia (+36.7%, *P* < 0.001) and combined anesthesia (+31.8%, *P* < 0.001), prolonged length of stay (6–7 days: +55.1%; 8–9 days: +63.6%; 10–42 days: +87.0%; all *P* < 0.001), gangrenous umbilical hernia (+118.0%, *P* = 0.013), and a higher number of additional diagnoses (1: +10.8%, *P* = 0.034; 2: +20.7%, *P* < 0.001; ≥3: +35.3%, *P* < 0.001).

**Conclusions:**

Hospitalization costs for umbilical hernia repair were significantly influenced by admission years, surgical approach, mesh use, ICU admission, length of hospital stay, anesthesia method, diagnostic complexity, and comorbidities. These findings provide important insights for clinicians and healthcare policymakers, highlighting potential areas for cost optimization, resource allocation, and policy interventions to reduce the economic burdens associated with umbilical hernia surgery.

## Introduction

Umbilical hernia is a common surgical condition characterized by protrusion of intra-abdominal contents through a defect in the abdominal wall around the umbilicus (1). Although often considered benign and straightforward, complications such as incarceration, strangulation, and gangrene may significantly increase the complexity and cost of clinical management (2). Given the aging population and the increased prevalence of obesity and other risk factors, the incidence and associated hospitalization burden of umbilical hernia are expected to rise, posing considerable challenges for healthcare systems (3, 4).

In recent decades, the escalating cost of healthcare and the growing burden of hospitalization expenses have drawn increasing attention globally. For common surgical conditions like umbilical hernia, understanding cost dynamics and the key factors driving hospitalization expenses is vital for health system planning and resource allocation. However, although clinical outcomes and surgical techniques have been extensively investigated, economic evaluations of umbilical and ventral hernia repairs remain relatively scarce worldwide, limiting the evidence base for cost-conscious decision-making in diverse healthcare systems (5, 6). This gap hinders effective strategies for cost control, optimization of surgical pathways, and informed policymaking.

Multiple demographic, clinical, and hospital-related factors can influence hospitalization costs, including patient demographics, clinical complexity, treatment modalities, surgical procedures, and perioperative management strategies (7, 8). Factors such as the surgical approach (open vs. laparoscopic), utilization of mesh materials, and the presence of additional comorbidities may substantially affect hospitalization costs (9–11). Previous international studies suggest considerable variability in cost components across demographic and clinical characteristics; nonetheless, comprehensive analyses based on large-scale data from China remain scarce.

In this context, we conducted a retrospective analysis over a 13-year period (2012–2024), utilizing hospital administrative data from patients diagnosed with umbilical hernia to evaluate hospitalization cost trends, analyze the distribution of different cost components, and systematically identify demographic, clinical, and management-related determinants of total hospitalization costs. By exploring cost composition and temporal trends, this study aims to elucidate the factors driving hospitalization expenses, providing essential insights for optimizing healthcare resources and enhancing efficiency. This comprehensive analysis can inform clinicians, hospital managers, and policymakers by highlighting key intervention points to control medical costs, improve economic sustainability, and enhance clinical outcomes in patients with umbilical hernia.

## Methods

### Data source

This study analyzed adult patients who underwent umbilical hernia repair surgery at Beijing Chaoyang Hospital, Capital Medical University, between January 2012 and December 2024. A total of 1,578 cases were included based on specific selection criteria. Patients were eligible if they (1) underwent umbilical hernia repair, (2) were 18 years or older, and (3) had complete medical records covering demographic information, hospitalization details, and cost-related data. Patients with incomplete medical records were excluded. All data were obtained from the hospital's electronic medical record system, specifically from the primary discharge records. This study was approved by the Ethics Committee of Beijing Chaoyang Hospital (Approval No. 2023-Ke-510).

### Data collection

Patient data included demographic details (gender, age, place of residence, marital status, and ethnicity), clinical characteristics [primary diagnosis, number of additional diagnoses, surgical approach, use of mesh, surgery grade, anesthesia method, intensive care unit (ICU) admission, ventilator use, and recurrence], and hospitalization details (admission year, length of stay, number of hospitalizations, admission route, and payment method). Age was categorized into four groups based on the quartiles of the cohort age distribution to obtain approximately balanced group sizes. Marital status was included as a sociodemographic variable because it may serve as a proxy for social support and healthcare utilization patterns. The primary outcome variable was total hospitalization cost, which was further categorized into diagnostic costs, treatment costs, nursing fees, medication expenses, biological product costs, material costs, and other associated expenses.

### Outcome Variables

The primary outcome was the total hospitalization cost per patient admission. Total hospitalization cost was defined as the billing-based total cost recorded in the hospital information system for a single admission (i.e., the amount charged/settled at discharge). All costs were reported in nominal CNY as recorded at discharge and were not adjusted for inflation or system-wide price reforms over 2012–2024. Costs were categorized into diagnosis, treatment, nursing, medication, biological products, materials, and other costs. These categories encompass major inpatient billing items, including nursing services; therapeutic procedures; diagnostic tests (e.g., pathology, laboratory testing, ultrasound, radiology); medications and biological products; single-use medical consumables (materials); and other miscellaneous fees (e.g., registration, and bed charges).

### Definition of key variables

Anesthesia method was extracted from the electronic anesthesia record (a structured field with predefined options). In our charting system, “combined anesthesia” is a selectable category indicating general anesthesia combined with a regional technique (e.g., spinal/epidural anesthesia and/or peripheral nerve block) for the same procedure.

Recurrence was ascertained from the hospital's electronic inpatient records. It was defined as either (1) documentation of recurrent umbilical hernia in the admission diagnosis/medical record at the index hospitalization, or (2) a subsequent inpatient admission for recurrent umbilical hernia managed within the same institution during the study period. Outpatient follow-up data and recurrence events managed at other hospitals were not comprehensively available; therefore, this variable captured only inpatient-documented, same-institution recurrence.

Surgical approach was defined based on operative records in the discharge dataset and categorized as open repair or laparoscopic repair. Open repair referred to hernia repair performed through a conventional abdominal wall incision with direct access to the hernia defect, including both primary suture repair and open mesh repair. Laparoscopic repair referred to hernia repair performed using trocar-based minimally invasive access with pneumoperitoneum and laparoscopic visualization. Robotic procedures were not performed or recorded in this cohort and therefore were not analyzed as a separate category.

Surgery grade was defined according to the Chinese national surgical classification standards (12):

- Grade 1 surgery refers to procedures that are low-risk, simple in process, and technically undemanding.
- Grade 2 surgery refers to procedures with certain risks, moderate procedural complexity, and some technical difficulty.
- Grade 3 surgery refers to procedures with higher risk, greater complexity, higher difficulty, and higher resource consumption.
- Grade 4 surgery refers to procedures with high risk, complex processes, high technical difficulty, high resource consumption, or major ethical concerns.

The specific definitions for each cost category are as follows:

- Nursing cost: Refers to costs related to nursing care services provided during hospitalization.
- Treatment cost: Includes costs associated with general therapeutic procedures such as physical therapy, radionuclide therapy, special treatment procedures, psychotherapy, interventional therapy, and rehabilitation therapy.
- Diagnosis cost: Refers to expenses for diagnostic procedures, including pathology, laboratory testing, radionuclide examination, ultrasound, and radiology services.
- Medication cost: Covers expenses related to drug use, including antimicrobial agents, Western medicines, traditional Chinese patent medicines, and herbal medicines.
- Biologicals cost: Refers specifically to costs on biological products, including albumin products, immunoglobulin products, coagulation factors, and cytokine preparations.
- Materials cost: Refers to the cost of single-use medical consumables, including those used for diagnostic procedures, therapeutic treatments, interventional procedures, and surgical operations.
- Other costs: Include expenses not categorized above, such as registration fees, bed charges, and miscellaneous hospital fees.

### Statistical analysis

All statistical analyses were performed using R version 4.3.3. Continuous variables were presented as mean ± standard deviation (SD), while categorical variables were expressed as frequency and percentage. Prior to regression analysis, we examined the distribution of hospitalization costs using descriptive statistics and histograms to assess normality. To evaluate group differences in raw cost data, we applied independent t-tests or Wilcoxon rank-sum tests for two-level categorical variables and one-way ANOVA or Kruskal–Wallis tests for variables with three or more levels, choosing the non-parametric option whenever normality was rejected (*P* < 0.05). Given the skewed distribution of cost data, a logarithmic (ln) transformation was applied to normalize the data.

For univariate analysis, linear regression models were constructed to assess the association between each independent variable and log-transformed hospitalization costs. The categorical variables included gender, age group, patient source, marital status, ethnicity, number of hospitalizations, payment method, admission route, admission year, length of hospital stay, primary diagnosis, number of additional diagnoses, surgical approach, mesh use, surgery grade, anesthesia method, ICU admission, ventilator use, and recurrence status. Models were fitted using the lm() function. Results are reported as regression coefficients (β), standard errors (SE), *P* values, and back-transformed percentage change in cost with 95% confidence intervals (CI). Percentage change relative to the reference group was calculated as: percentage change (%) = 100 × [exp(β) − 1], with the corresponding 95% CI calculated as: 95% CI (%) = 100 × [exp(CI_low) − 1] to 100 × [exp(CI_high) − 1].

For the multivariable analysis, a prespecified full multivariable linear regression model was fitted with log-transformed total hospitalization cost as the dependent variable. Covariates were selected *a priori* based on clinical relevance and data availability, and all prespecified variables were entered simultaneously using a forced-entry approach. Back-transformed effects are presented as adjusted percentage change in cost (with 95% CI) using the same transformation described above. To address the clinical correlation between surgical approach and mesh use, sensitivity analyses were conducted using stratified multivariable models: the multivariable regression was repeated separately within the open-repair subgroup and the laparoscopic-repair subgroup, using the same covariate set as the primary model but excluding surgical approach within each stratum.

Model diagnostics included examination of residual plots to assess model fit, identification of influential observations using Cook's distance, and evaluation of multicollinearity using the variance inflation factor (VIF). The VIF values were calculated to ensure that multicollinearity was within acceptable limits (VIF < 10). To evaluate whether the associations of key cost drivers changed over time, prespecified interaction terms were added to the multivariable log-linear model by including cross-product terms between admission period, surgical approach, and mesh use. Overall interaction effects were assessed using nested-model comparisons with *F* tests. Period-specific adjusted effects were derived from the fitted interaction models and reported as percentage change in cost, with 95% confidence intervals obtained by back-transforming the relevant coefficients.

For international readability, we additionally provide approximate USD and EUR equivalents for selected key cost estimates, converted using the 2025 annual average exchange rates (1 USD = 7.1429 CNY; 1 EUR = 8.0965 CNY) (13). These conversions are presented for reference only and do not account for purchasing power parity. All primary analyses were performed in CNY.

A two-sided *P*-value of <0.05 was considered statistically significant.

## Results

### Clinical and demographic characteristics of patients

**Figure 1:**
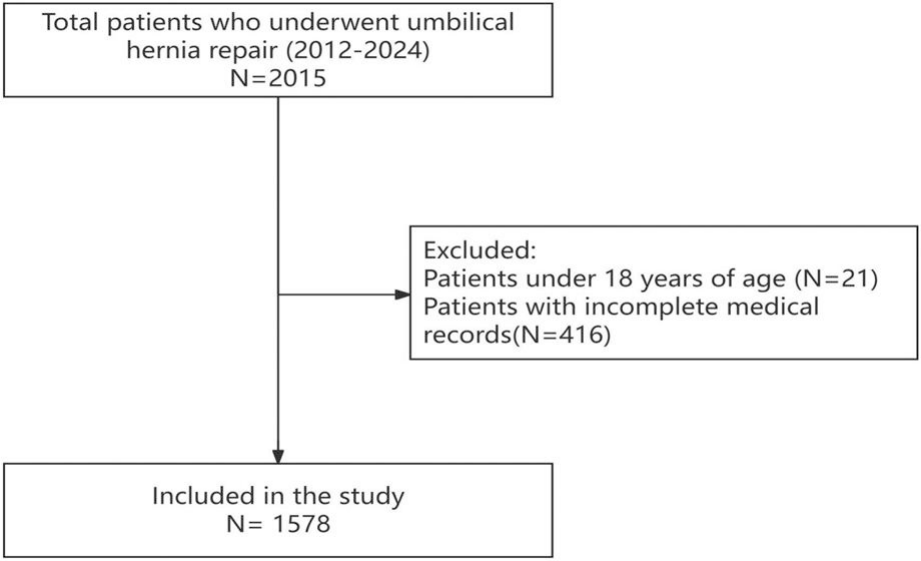
Flowchart of patient selection for umbilical hernia repair (2012–2024).

A total of 2,015 patients who underwent umbilical hernia repair surgery between 2012 and 2024 were initially identified. After exclusion of 21 patients younger than 18 years and 416 patients with incomplete medical records, 1,578 adult patients were included in the final analysis (Figure 1, Table 1). The mean total hospitalization cost was 32,218.17 ± 18,624.60 CNY (≈USD 4,511; ≈EUR 3,979). Among these patients, 843 (53.42%) were female, and 735 (46.58%) were male, with female patients incurring significantly higher hospitalization costs compared to male patients [34,478.98 ± 19,032.68 CNY [≈USD 4,827; ≈EUR 4,259] vs. 29,625.16 ± 17,807.77 CNY [≈USD 4,147; ≈EUR 3,659], *P* < 0.001]. The majority were residents of Beijing (80.23%), married (95.31%), and of Han ethnicity (94.74%). Patients who underwent laparoscopic surgery [40,129.10 ± 16,468.19 CNY (≈USD 5,618; ≈EUR 4,956)] incurred significantly higher costs compared to those who underwent open surgery [17,514.15 ± 12,455.71 CNY (≈USD 2,452; ≈EUR 2,163), *P* < 0.001]. ICU admission [54,174.03 ± 23,644.98 CNY [≈USD 7,584; ≈EUR 6,691] vs. 31,380.38 ± 17,889.44 CNY [≈USD 4,393; ≈EUR 3,876], *P* < 0.001] and ventilator use [62,018.84 ± 22,563.05 CNY [≈USD 8,683; ≈EUR 7,660] vs. 31,581.65 ± 18,009.78 CNY [≈USD 4,421; ≈EUR 3,901], *P* < 0.001] were also associated with significantly increased hospitalization costs. Patients experiencing recurrence had higher mean costs [41,605.56 ± 20,070.09 CNY (≈USD 5,825; ≈EUR 5,139)] than those without recurrence [32,176.34 ± 18,614.20 CNY (≈USD 4,505; ≈EUR 3,974)], though the difference was not statistically significant (*P* = 0.181). Additionally, factors such as number of hospitalizations (*P* < 0.001), payment method (*P* = 0.036), admission route (*P* = 0.006), primary diagnosis (*P* < 0.001), mesh use (*P* < 0.001), primary surgery grade (*P* < 0.001), anesthesia method (*P* < 0.001), number of additional diagnoses (*P* < 0.001), and length of hospital stay (*P* < 0.001) significantly influenced hospitalization costs.

**Table 1:** Clinical and demographic characteristics of patients with umbilical hernia.

Variable	Group	Cases (*N* = 1,578)	Proportion (%)	Average total hospitalization cost (CNY, mean ± SD)	*P* value
Gender	Male	735	46.58	29,625.16 ± 17,807.77	<0.001
	Female	843	53.42	34,478.98 ± 19,032.68	
Age group	18–41	396	25.10	32,424.24 ± 20,106.65	0.908
	42–58	405	25.67	32,168.53 ± 17,472.64	
	59–70	413	26.17	32,258.67 ± 17,250.93	
	71–96	364	23.07	32,003.25 ± 19,733.12	
Admission year	2012–2015	182	11.53	24,499.33 ± 15,355.74	<0.001
	2016–2019	497	31.50	34,569.13 ± 16,583.03	
	2020–2024	899	56.97	32,481.13 ± 19,862.90	
Patient source	Beijing	1,266	80.23	32,005.50 ± 18,454.79	0.441
	Other Provinces	312	19.77	33,081.09 ± 19,304.69	
Marital status	Unmarried	74	4.69	34,295.85 ± 21,774.11	0.489
	Married	1,504	95.31	32,115.94 ± 18,458.16	
Ethnicity	Han	1,495	94.74	32,147.07 ± 18,734.14	0.469
	Minority	83	5.26	33,498.72 ± 16,576.95	
Number of hospitalizations	1 time	1,414	89.61	32,883.15 ± 18,674.45	<0.001
	2 times	116	7.35	27,843.33 ± 17,438.28	
	≥3 times	48	3.04	23,201.47 ± 16,335.42	
Payment method	Non-medical insurance	195	12.36	34,710.99 ± 18,694.36	0.036
	Medical insurance	1,383	87.64	31,866.69 ± 18,594.65	
Admission route	Emergency	146	9.25	28,967.00 ± 20,992.75	0.006
	Outpatient	1,432	90.75	32,549.64 ± 18,341.84	
Length of hospital stay (days)	1–5	519	32.89	20,361.10 ± 15,598.95	<0.001
	6–7	378	23.95	37,010.86 ± 16,151.14	
	8–9	306	19.39	39,306.45 ± 16,383.80	
	10–42	375	23.76	38,013.28 ± 18,725.97	
Primary diagnosis	Umbilical hernia	1,438	91.13	32,567.27 ± 18,195.12	<0.001
	Incarcerated umbilical hernia	58	3.68	26,056.06 ± 22,786.29	
	Umbilical hernia with obstruction	80	5.07	29,709.82 ± 21,711.00	
	Gangrenous umbilical hernia	2	0.13	60,246.23 ± 972.70	
Number of additional diagnoses	0	145	9.19	22,483.11 ± 16,691.73	<0.001
	1	173	10.96	29,162.97 ± 17,510.90	
	2	221	14.01	31,154.07 ± 17,843.31	
	≥3	1,039	65.84	34,311.81 ± 18,725.18	
Surgery approach	Open	552	34.98	17,514.15 ± 12,455.71	<0.001
	Laparoscopic	1,026	65.02	40,129.10 ± 16,468.19	
Mesh use	No	137	8.68	13,355.73 ± 11,364.61	<0.001
	Yes	1,441	91.32	34,011.47 ± 18,182.72	
Primary surgery grade	Grade 2	119	7.54	14,995.15 ± 11,267.94	<0.001
	Grade 3	1,244	78.83	32,900.92 ± 18,766.86	
	Grade 4	215	13.62	37,800.46 ± 15,540.69	
Primary anesthesia method	Local anesthesia	256	16.22	13,577.68 ± 8,445.89	<0.001
	General anesthesia	1,277	80.93	36,450.06 ± 17,808.46	
	Combined anesthesia	45	2.85	18,170.26 ± 8,866.50	
Intensive care unit	No	1,520	96.32	31,380.38 ± 17,889.44	<0.001
	Yes	58	3.68	54,174.03 ± 23,644.98	
Ventilator use	No	1,545	97.91	31,581.65 ± 18,009.78	<0.001
	Yes	33	2.09	62,018.84 ± 22,563.05	
Recurrence	No	1,571	99.56	32,176.34 ± 18,614.20	0.181
	Yes	7	0.44	41,605.56 ± 20,070.09	

Age categories were defined using cohort-specific quartiles to obtain approximately balanced group sizes (cut-points at the 25th, 50th, and 75th percentiles), resulting in 18–41, 42–58, 59–70, and 71–96 years; USD/EUR equivalents are approximate and were calculated using the 2025 annual average exchange rates (1 USD = 7.1429 CNY; 1 EUR = 8.0965 CNY).

### Trends in hospitalization costs and cost composition

**Figure 2:**
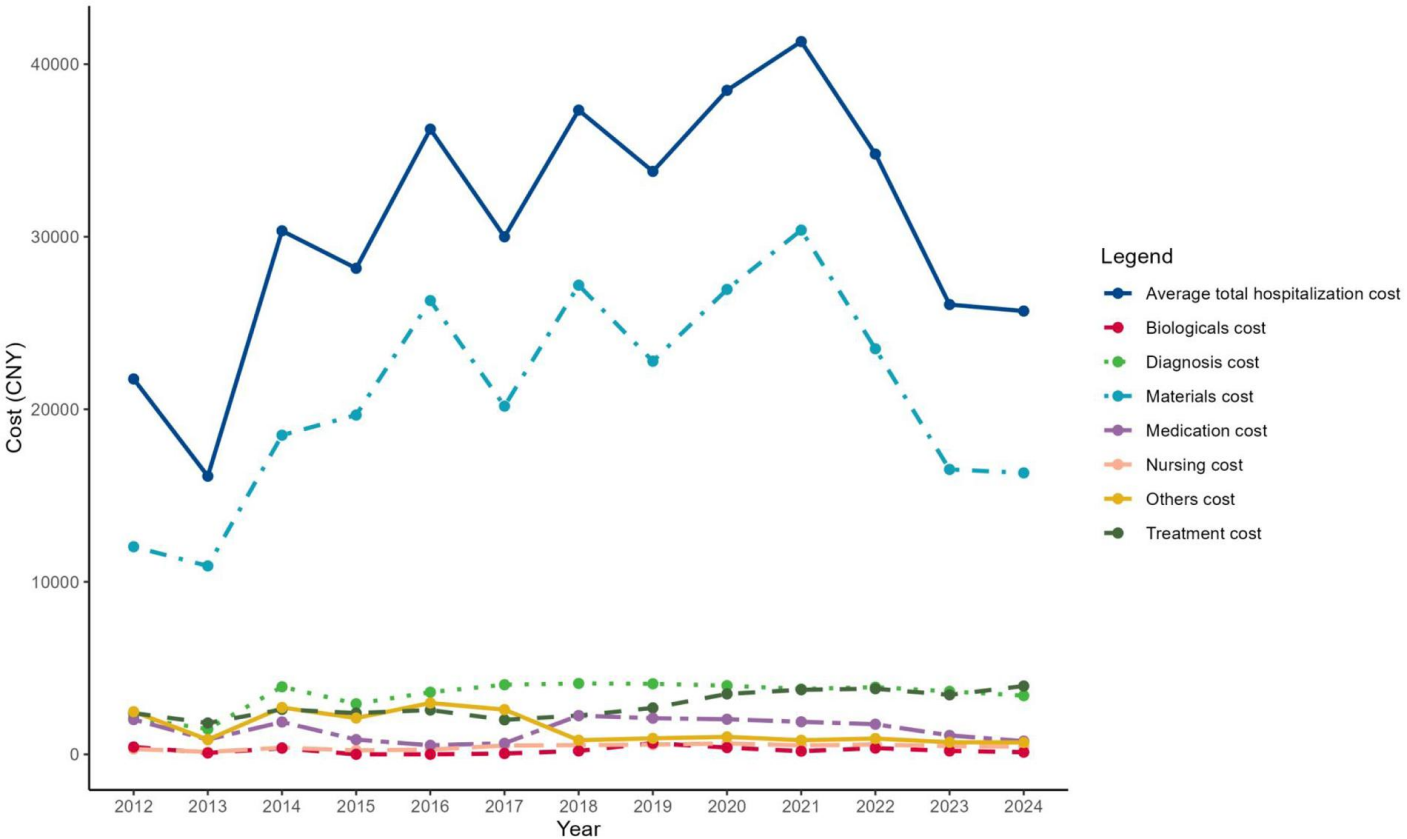
Trends in hospitalization costs and cost composition for patients with umbilical hernia (2012–2024).

From 2012 to 2024, hospitalization costs showed an overall upward trend, increasing from 21,756.86 CNY (≈USD 3,046; ≈EUR 2,687) in 2012 to a peak of 41,314.14 CNY (≈USD 5,784; ≈EUR 5,103) in 2021, followed by a decline to 25,692.89 CNY (≈USD 3,597; ≈EUR 3,173) in 2024 (Table 2, Figure 2). Cost composition varied considerably across years. Material costs consistently accounted for the highest proportion, ranging from 55.30% in 2012 to a peak of 73.55% in 2021. Medication expenses showed a decreasing trend over the study period, declining from 9.26% in 2012 to 2.98% in 2024. Treatment and diagnostic costs remained relatively stable, with slight annual fluctuations.

**Table 2:** Trends in hospitalization costs and cost composition for patients with umbilical hernia (2012–2024).

Year	Average total hospitalization cost	Average daily hospitalization cost	Diagnosis		Treatment		Nursing		Medication		Biologicals		Materials		Others	
			Cost	Proportion (%)	Cost	Proportion(%)	Cost	Proportion (%)	Cost	Proportion (%)	Cost	Proportion (%)	Cost	Proportion (%)	Cost	Proportion (%)
2012	21,756.86	2,334.45	2,112.83	9.71	2,390.23	10.99	310.76	1.43	2,015.34	9.26	424.35	1.95	12,031.73	55.30	2,471.62	11.36
2013	16,123.14	2,793.95	1,467.11	9.10	1,814.36	11.25	135.29	0.84	855.21	5.30	79.71	0.49	10,919.17	67.72	852.29	5.29
2014	30,344.4	4,007.39	3,914.14	12.90	2,605.10	8.59	384.93	1.27	1,874.64	6.18	355.43	1.17	18,497.21	60.96	2,712.96	8.94
2015	28,173.63	3,746.71	2,931.78	10.41	2,392.37	8.49	229.3	0.81	852.22	3.02	0.00	0.00	19,666.3	69.80	2,101.66	7.46
2016	36,238.46	4,261.48	3,601.46	9.94	2,567.42	7.08	271.76	0.75	523.33	1.44	0.00	0.00	26,300.72	72.58	2,973.78	8.21
2017	29,999.34	3,713.65	4,033.34	13.44	1,999.98	6.67	506.80	1.69	640.82	2.14	46.67	0.16	20,181.64	67.27	2,590.08	8.63
2018	37,339.35	5,101.9	4,106.68	11.00	2,245.67	6.01	536.65	1.44	2,243.73	6.01	196.00	0.52	27,192.30	72.82	818.31	2.19
2019	33,788.34	4,764.12	4,086.50	12.09	2,689.40	7.96	564.25	1.67	2,092.24	6.19	645.78	1.91	22,784.71	67.43	925.46	2.74
2020	38,489.55	6,379.82	3,978.86	10.34	3,500.89	9.10	627.61	1.63	2,032.34	5.28	389.27	1.01	26,948.59	70.02	1,012.00	2.63
2021	41,314.14	7,148.46	3,783.95	9.16	3,741.68	9.06	520.19	1.26	1,886.37	4.57	180.62	0.44	30,384.89	73.55	816.43	1.98
2022	34,793.81	6,553.00	3,893.35	11.19	3,802.65	10.93	571.50	1.64	1,744.09	5.01	361.16	1.04	23,507.07	67.56	914.00	2.63
2023	26,069.67	5,777.70	3,645.62	13.98	3,447.36	13.22	469.44	1.80	1,097.33	4.21	196.21	0.75	16,513.62	63.34	700.09	2.69
2024	25,692.89	5,469.74	3,402.50	13.24	3,958.67	15.41	441.81	1.72	764.85	2.98	125.18	0.49	16,313.52	63.49	686.36	2.67

USD/EUR equivalents are approximate and were calculated using the 2025 annual average exchange rates (1 USD = 7.1429 CNY; 1 EUR = 8.0965 CNY).

### Factors associated with hospitalization costs

In the univariate regression analysis, higher total hospitalization costs were significantly associated with female sex (+19.3%, *P* < 0.001), later admission years (2016–2019: +44.2%, *P* < 0.001; 2020–2024: +25.2%, *P* < 0.001), outpatient admission (+16.3%, *P* = 0.019), longer length of stay (6–7 days: +118.3%; 8–9 days: +134.9%; 10–42 days: +123.4%; all *P* < 0.001), laparoscopic repair (+145.2%, *P* < 0.001), mesh use (+176.7%, *P* < 0.001), higher surgery grade (Grade 3: +130.6%; Grade 4: +198.8%; both *P* < 0.001), general anesthesia (+179.3%, *P* < 0.001) or combined anesthesia (+45.2%, *P* < 0.001), ICU admission (+93.8%, *P* < 0.001), ventilator use (+129.4%, *P* < 0.001), and an increasing number of additional diagnoses (1: +42.0%; 2: +54.2%; ≥3: +77.4%; all *P* < 0.001). In contrast, lower costs were observed among patients with multiple hospitalizations (2 times: −17.9%, *P* = 0.006; ≥3 times: −30.2%, *P* = 0.001) and those with incarcerated umbilical hernia (−24.3%, *P* = 0.005) (Table 3).

**Table 3:** Univariate analysis of factors associated with total hospitalization costs in patients with umbilical hernia.

Variable	Group	Log-transformed individual total hospitalization costs (mean ± SD)	β	Standard error	% change in cost	95% CI	*P* value
Gender	Male	10.06 ± 0.75	Ref.				
	Female	10.24 ± 0.73	0.176	0.037	19.3	(10.9–28.3)	<0.001
Age group	18–41	10.12 ± 0.83	Ref.				
	42–58	10.17 ± 0.71	0.057	0.053	5.9	(−4.5–17.4)	0.275
	59–70	10.19 ± 0.69	0.077	0.052	8.0	(−2.5–19.7)	0.140
	71–96	10.14 ± 0.75	0.027	0.054	2.7	(−7.6–14.2)	0.620
Admission year	2012–2015	9.91 ± 0.67	Ref.				
	2016–2019	10.28 ± 0.66	0.366	0.064	44.2	(27.2–63.4)	<0.001
	2020–2024	10.14 ± 0.79	0.225	0.060	25.2	(11.3–40.8)	<0.001
Patient source	Beijing	10.15 ± 0.74	Ref.				
	Other Provinces	10.18 ± 0.74	0.035	0.047	3.6	(−5.6–13.6)	0.457
Marital status	Unmarried	10.19 ± 0.79	Ref.				
	Married	10.16 ± 0.74	−0.032	0.089	−3.2	(−18.6–15.2)	0.715
Ethnicity	Han	10.15 ± 0.75	Ref.				
	Minority	10.25 ± 0.66	0.097	0.084	10.2	(−6.5–29.9)	0.248
Number of hospitalizations	1 time	10.18 ± 0.74	Ref.				
	2 times	9.98 ± 0.79	−0.198	0.071	−17.9	(−28.7–−5.6)	0.006
	≥3 times	9.82 ± 0.69	−0.360	0.109	−30.2	(−43.6–−13.7)	0.001
Payment method	Non-medical insurance	10.24 ± 0.75	Ref.				
	Medical insurance	10.14 ± 0.74	−0.096	0.057	−9.2	(−18.8–1.5)	0.091
Admission route	Emergency	10.02 ± 0.73	Ref.				
	Outpatient	10.17 ± 0.74	0.151	0.065	16.3	(2.5–32.0)	0.019
Length of hospital stay (days)	1–5	9.61 ± 0.82	Ref.				
	6–7	10.39 ± 0.55	0.781	0.043	118.3	(100.6–137.7)	<0.001
	8–9	10.47 ± 0.51	0.854	0.046	134.9	(114.6–157.1)	<0.001
	10–42	10.42 ± 0.53	0.804	0.043	123.4	(105.2–143.2)	<0.001
Primary diagnosis	Umbilical hernia	10.17 ± 0.74	Ref.				
	Incarcerated umbilical hernia	9.89 ± 0.73	−0.278	0.099	−24.3	(−37.7–−8.0)	0.005
	Umbilical hernia with obstruction	10.05 ± 0.71	−0.119	0.085	−11.2	(−24.9–4.9)	0.162
	Gangrenous umbilical hernia	11.01 ± 0.02	0.834	0.525	130.3	(−17.7–544.6)	0.112
Number of additional diagnoses	0	9.68 ± 0.9	Ref.				
	1	10.03 ± 0.79	0.351	0.082	42.0	(21.0–66.7)	<0.001
	2	10.11 ± 0.77	0.433	0.077	54.2	(32.5–79.5)	<0.001
	≥3	10.25 ± 0.67	0.573	0.064	77.4	(56.4–101.2)	<0.001
surgical approach	Open	9.57 ± 0.65	Ref.				
	Laparoscopic	10.47 ± 0.59	0.897	0.032	145.2	(130.3–161.2)	<0.001
Mesh use	No	9.23 ± 0.73	Ref.				
	Yes	10.25 ± 0.68	1.018	0.061	176.7	(145.3–212.1)	<0.001
Primary surgery grade	Grade 2	9.35 ± 0.78	Ref.				
	Grade 3	10.18 ± 0.73	0.836	0.067	130.6	(102.1–163.2)	<0.001
	Grade 4	10.44 ± 0.47	1.095	0.080	198.8	(155.3–249.8)	<0.001
Primary anesthesia method	Local anesthesia	9.31 ± 0.69	Ref.				
	General anesthesia	10.34 ± 0.63	1.027	0.044	179.3	(156.4–204.2)	<0.001
	Combined anesthesia	9.69 ± 0.52	0.373	0.103	45.2	(18.7–77.7)	<0.001
Intensive care unit	No	10.13 ± 0.74	Ref.				
	Yes	10.79 ± 0.49	0.662	0.098	93.8	(59.9–134.9)	<0.001
Ventilator use	No	10.14 ± 0.74	Ref.				
	Yes	10.97 ± 0.38	0.830	0.129	129.4	(78.1–195.6)	<0.001

Percentage change in cost was calculated from the log-linear model as [exp(β) − 1] × 100 and represents the percent change in total hospitalization cost relative to the reference group.

Multivariable linear regression analysis identified several independent factors significantly associated with increased total hospitalization costs (Table 4), and multicollinearity was low across covariates (all VIFs ≤ 1.80; Supplementary Table S1). The model demonstrated good explanatory performance, with an adjusted R-squared of 0.68 on the log-cost scale. Compared with admissions in 2012–2015, later admission years were associated with higher costs (2016–2019: +30.4%, 95% CI 20.7%–41.0%; *P* < 0.001; 2020–2024: +45.4%, 95% CI 34.5%–57.2%; *P* < 0.001). Laparoscopic repair (+107.9%, 95% CI 95.2%–121.3%; *P* < 0.001) and mesh use (+108.2%, 95% CI 91.7%–126.1%; *P* < 0.001) were also associated with higher costs. Prolonged length of stay showed a strong graded association with costs (6–7 days: +55.1%; 8–9 days: +63.6%; 10–42 days: +87.0%; all *P* < 0.001). Additional independent predictors included ICU admission (+68.6%, 95% CI 40.8%–101.9%; *P* < 0.001), general anesthesia (+36.7%, 95% CI 26.8%–47.4%; *P* < 0.001) or combined anesthesia (+31.8%, 95% CI 15.2%–50.8%; *P* < 0.001), gangrenous umbilical hernia (+118.0%, 95% CI 18.1%–302.5%; *P* = 0.013), and a higher number of additional diagnoses (1: +10.8%, *P* = 0.034; 2: +20.7%, *P* < 0.001; ≥3: +35.3%, *P* < 0.001).

**Table 4:** Multivariate analysis of factors associated with total hospitalization costs in patients with umbilical hernia.

Variable	Group	β	Standard error	% change in cost	95% CI	*P* value
Gender	Male	Ref.				
	Female	−0.006	0.024	−0.6	(−5.2–4.2)	0.811
Age group	18–41	Ref.				
	42–58	0.021	0.031	2.1	(−4.0–8.5)	0.508
	59–70	−0.023	0.034	−2.2	(−8.6–4.6)	0.512
	71–96	−0.007	0.037	−0.7	(−7.7–6.8)	0.853
Admission year	2012–2015	Ref.				
	2016–2019	0.266	0.04	30.4	(20.7–41.0)	<0.001
	2020–2024	0.374	0.04	45.4	(34.5–57.2)	<0.001
Patient source	Beijing	Ref.				
	Other Provinces	−0.005	0.03	−0.5	(−6.2–5.5)	0.866
Marital status	Unmarried	Ref.				
	Married	−0.041	0.052	−4.1	(−13.3–6.2)	0.425
Ethnicity	Han	Ref.				
	Minority	0.052	0.048	5.3	(−4.2–15.7)	0.283
Number of hospitalizations	1 time	Ref.				
	2 times	−0.042	0.042	−4.1	(−11.6–4.1)	0.315
	≥3 times	−0.020	0.063	−2	(−13.4–11)	0.750
Payment method	Non-medical insurance	Ref.				
	Medical insurance	−0.057	0.036	−5.6	(−12.1–1.4)	0.117
Admission route	Emergency	Ref.				
	Outpatient	−0.019	0.066	−1.9	(−13.8–11.6)	0.767
Length of hospital stay (days)	1–5	Ref.				
	6–7	0.439	0.031	55.1	(46.0–64.7)	<0.001
	8–9	0.492	0.034	63.6	(53.1–74.8)	<0.001
	10–42	0.626	0.033	87.0	(75.5–99.4)	<0.001
Primary diagnosis	Umbilical hernia	Ref.				
	Incarcerated umbilical hernia	−0.083	0.076	−8	(−20.8–6.8)	0.272
	Umbilical hernia with obstruction	0.113	0.078	12	(−4.0–30.6)	0.149
	Gangrenous umbilical hernia	0.779	0.313	118.0	(18.1–302.5)	0.013
Number of additional diagnoses	0	Ref.				
	1	0.103	0.048	10.8	(0.8–21.8)	0.034
	2	0.188	0.046	20.7	(10.2–32.2)	<0.001
	≥3	0.303	0.042	35.3	(24.7–46.9)	<0.001
surgical approach	Open	Ref.				
	Laparoscopic	0.732	0.032	107.9	(95.2–121.3)	<0.001
Mesh use	No	Ref.				
	Yes	0.733	0.042	108.2	(91.7–126.1)	<0.001
Primary surgery grade	Grade 2	Ref.				
	Grade 3	−0.077	0.048	−7.4	(−15.7–1.6)	0.105
	Grade 4	−0.011	0.057	−1.1	(−11.6–10.6)	0.843
Primary anesthesia method	Local anesthesia	Ref.				
	General anesthesia	0.313	0.038	36.7	(26.8–47.4)	<0.001
	Combined anesthesia	0.276	0.069	31.8	(15.2–50.8)	<0.001
Intensive care unit	No	Ref.				
	Yes	0.522	0.092	68.6	(40.8–101.9)	<0.001
Ventilator use	No	Ref.				
	Yes	−0.048	0.114	−4.7	(−23.7–19.1)	0.672

Percentage change in cost was calculated from the log-linear model as [exp(β) − 1] × 100 and represents the percent change in total hospitalization cost relative to the reference group.

In interaction analyses assessing temporal changes in key cost drivers (Supplementary Table S2), significant effect modification by admission period was observed for both surgical approach (admission period × surgical approach: F = 4.525, *P* = 0.011) and mesh use (admission period × mesh use: F = 17.909, *P* < 0.001). The adjusted cost difference associated with laparoscopic vs. open repair increased from +68.1% (95% CI, 44.3%–95.8%) in 2012–2015 to +112.7% (95% CI, 94.0%–133.2%) in 2016–2019 and remained high in 2020–2024 (+110.7%, 95% CI, 96.0%–126.5%) (all *P* < 0.001). For mesh use, the period-specific association strengthened over time: mesh use was not significantly associated with higher costs in 2012–2015 (+21.0%, 95% CI, −7.4% to 58.2%; *P* = 0.162), but was associated with substantially higher costs in 2016–2019 (+71.4%, 95% CI, 48.7%–97.6%; *P* < 0.001) and 2020–2024 (+150.1%, 95% CI, 125.4%–177.4%; *P* < 0.001).

Sensitivity analyses stratified by surgical approach showed that mesh use was consistently associated with higher total hospitalization costs within each stratum (Supplementary Tables S3–S4). In the open repair subgroup, mesh use was associated with a 50.7% higher total cost compared with non-mesh repair (95% CI, 33.2%–70.5%; *P* < 0.001). In the laparoscopic repair subgroup, mesh use was associated with a 180.3% higher total cost (95% CI, 151.8%–212.0%; *P* < 0.001).

## Discussion

In this retrospective study of 1,578 patients undergoing umbilical hernia repair between 2012 and 2024, we identified several critical factors associated with hospitalization costs. Overall, the hospitalization expenses demonstrated an increasing trend, peaking in 2021 and subsequently decreasing slightly. The predominant cost component was surgical materials, whose proportion consistently increased over the study period, reflecting shifts in healthcare policy and clinical practice. The multivariate analysis identified laparoscopic surgery, mesh use, prolonged hospital stay, later admission year, ICU admission, advanced anesthesia methods, complicated primary diagnoses, and additional comorbidities as significant independent predictors of higher hospitalization costs.

### Overall trends in hospitalization costs

The upward trend in total hospitalization costs until 2021 may reflect multiple factors, including changes in surgical practice, greater use of specialized materials and disposable consumables, and broader increases in healthcare costs over time. The concurrent rise in the proportion of material costs and decline in medication costs may be consistent with evolving policy and practice environments in China (14, 15). In the present study, material costs mainly represent costs of single-use medical consumables, which are closely related to the operative approach and the intensity of device and consumable use. The increase in material costs over time may therefore partly reflect greater adoption of minimally invasive techniques and increased use of procedure-specific disposable items. However, because the discharge-record data only provide aggregated billing categories rather than itemized charges, the specific consumables underlying these trends could not be identified directly. The decline in total costs after 2021 may also be related to recent healthcare cost-control measures, including centralized procurement and tighter regulation of medical consumables, although this interpretation should be regarded as contextual rather than causal (16, 17).

A further consideration is the potential influence of the COVID-19 pandemic on in-hospital resource use and cost structure, particularly within the materials category. During the pandemic, infection-prevention requirements may have increased the use of personal protective equipment and other single-use items across perioperative and inpatient care, while supply-chain disruptions may also have affected the costs of disposable materials (18, 19). In addition, changes in perioperative workflows, screening procedures, staffing patterns, surgical volume, and case mix during this period may have indirectly influenced care intensity and consumable use (20, 21). For higher-acuity patients, infection-control practices in intensive care settings may also have contributed to greater use of disposable devices and supplies (22). Taken together, these factors may help contextualize the temporal fluctuations observed in hospitalization costs, especially material costs, but their individual contributions cannot be quantified from the available data.

### Factors influencing hospitalization costs

Admission period remained independently associated with higher in-hospital costs after adjustment and may reflect a composite “system-level” signal rather than a purely temporal trend. The 2012–2015 baseline period preceded or overlapped with early phases of China's public-hospital reforms, whereas 2016–2019 coincided with intensified policies to curb pharmaceutical spending (e.g., zero-markup drug reforms), which have been associated with reduced drug costs but also shifts toward non-drug cost components such as services, materials, and diagnostics (23–25). During 2020–2024, the hospital cost structure may have been further influenced by pandemic-era infection-prevention and ICU preparedness practices, as well as ongoing payment and purchasing reforms (e.g., DRG/DIP payment pilots and broader centralized procurement), all of which could alter both provider incentives and the relative contribution of materials vs. other cost components (19, 26–28). Within this context, the significant period × surgical-factor interactions (Supplementary Table S2) suggest that the cost associations of laparoscopy and, more notably, mesh use were not stable across periods. This pattern is consistent with evolving practice patterns and procurement/payment environments in which these variables may increasingly reflect the intensity of device and consumable utilization captured in billing, rather than a fixed technology-related cost premium over time (27, 28).

Among the significant predictors, laparoscopic surgery was strongly associated with higher hospitalization costs. Although laparoscopic techniques have recognized clinical advantages, such as fewer wound complications and faster postoperative recovery, they also involve additional equipment and disposable material costs. Previous studies have similarly reported higher hospitalization costs for laparoscopic procedures because of the greater expense of minimally invasive surgical devices (7, 29). Thus, our findings suggest that the choice of surgical approach may be interpreted in light of both expected clinical benefits and in-hospital cost implications.

Similarly, mesh use was consistently associated with higher hospitalization costs in this cohort. Mesh repair is well-documented to reduce recurrence rates and improve clinical outcomes. However, the economic implications associated with the universal application of mesh materials remain a substantial burden to patients and healthcare systems. Evidence suggests that, while mesh repair may reduce recurrence, its incremental benefits in other patient-important outcomes (e.g., postoperative pain, wound complications, or quality of life) are less consistent raising concerns about whether routine mesh use represents good value given its additional device-related costs (30, 31). Importantly, mesh use and surgical approach are closely linked in clinical practice—particularly because laparoscopic repair is commonly mesh-based—and procedure-related material consumption differs substantially between approaches. Sensitivity analyses stratified by surgical approach showed that the association between mesh use and higher total costs persisted within both open and laparoscopic strata (Supplementary Tables S3–S4), supporting the interpretation that mesh use captures additional material/resource inputs beyond surgical approach alone. The observed cost difference in the laparoscopic subgroup (a ∼2.8-fold increase) likely reflects, at least in part, unmeasured case complexity and differences in material/device utilization (e.g., fixation devices and laparoscopic disposables) that are not fully captured in the administrative dataset. Therefore, mesh placement decisions may need to take into account clinical indications and individual patient characteristics, in addition to potential cost implications.

Interpretation of the associations for laparoscopic repair and mesh use should also explicitly consider confounding by indication. In routine practice, selection of a minimally invasive approach and the decision to implant mesh are closely tied to baseline patient risk and operative complexity—factors such as hernia defect size or classification, obesity status (BMI), ASA physical status, and perioperative/postoperative morbidity (e.g., complications)—which can simultaneously influence resource utilization, length of stay, ICU needs, and total costs. Because these clinical severity and outcome measures were not available in the discharge-record dataset, residual confounding is likely, and the observed cost differentials for laparoscopy and mesh use may partly reflect underlying case complexity and downstream care intensity rather than the procedural choices alone.

Length of stay (LOS) showed a strong association with total hospitalization costs, consistent with prior studies (7, 8). Importantly, LOS is a downstream measure of care utilization that likely integrates multiple clinical processes, including baseline patient complexity, perioperative management, and postoperative morbidity. In the absence of standardized complication measures (e.g., Clavien–Dindo grading), a prolonged LOS should be interpreted primarily as a marker of higher care intensity and potential postoperative events rather than an isolated determinant of cost. From a practical perspective, optimizing perioperative pathways may help mitigate potentially avoidable variation in LOS. Enhanced recovery after surgery (ERAS) programs have been associated with shorter LOS without compromising outcomes in suitable surgical populations (32, 33). In this context, ERAS-based pathway standardization and timely discharge planning may be relevant when interpreting opportunities to improve efficiency in hernia care, while recognizing that a proportion of prolonged hospitalizations will remain clinically appropriate due to patient severity and postoperative course.

Similarly, ICU admission was associated with higher hospitalization costs and likely reflects a subset of patients with greater perioperative severity, complications, or monitoring requirements. ICU care is inherently resource-intensive, requiring continuous monitoring, specialized staffing, and advanced interventions, which plausibly contribute to higher costs (34, 35). However, ICU use may also be influenced by institutional practice patterns and perioperative risk tolerance, which are not fully captured in administrative data. Therefore, ICU admission in this study may be better understood as a marker of high-acuity care and resource use, rather than being interpreted solely as an independent driver of costs. These observations highlight the need for future studies incorporating standardized perioperative risk measures and postoperative complication data to more rigorously evaluate determinants of ICU utilization and the appropriateness of ICU triage strategies.

We also identified general or combined anesthesia as predictors of higher hospitalization costs compared to local anesthesia. While general anesthesia ensures patient comfort and optimal surgical conditions in complex cases, it significantly raises costs due to increased anesthesia-related staffing, equipment, and monitoring (36, 37). Given this, the use of general anesthesia for elective hernia repair may warrant consideration in relation to clinical necessity, with local or regional approaches potentially suitable in selected cases.

The association between a greater number of additional diagnoses and higher hospitalization costs highlights the substantial influence of clinical complexity on healthcare costs. In our study, however, not all complicated primary diagnoses were associated with increased costs to the same extent. Although complex hernias may require more intensive management, incarcerated umbilical hernia was not independently associated with higher costs after adjustment, whereas gangrenous umbilical hernia was associated with substantially increased costs, likely reflecting greater perioperative complexity, longer monitoring, and more intensive resource use. These findings suggest that cost escalation may be driven more by severe complicated presentations, rather than by incarceration alone. This pattern is consistent with the potential value of early diagnosis and timely surgical management in reducing progression to more severe stages, although this hypothesis warrants further evaluation in studies specifically designed for that purpose.

### Recurrence and hospitalization costs

In our study, recurrence was uncommon (7/1,578, 0.44%) and was identified only from inpatient records within the same institution, so outpatient recurrences or those managed elsewhere may not have been captured. Patients with documented recurrence had higher mean hospitalization costs than those without recurrence (41,605.56 vs. 32,176.34 CNY), although this observation was based on a small number of recurrent cases. Contemporary series in which mesh repair predominates report recurrence rates of 2%–6%—for example, Shankar et al. reported 2.4% after mesh vs. 9.8% after primary suture repair, and meta-analyses similarly suggest lower recurrence with mesh than with suture closure (31, 38). Economic studies further indicate that admissions for recurrent hernia are more costly than index repairs—approximately 10%–20% higher in some reports—and that additional spending may be amplified in the presence of modifiable risk factors (e.g., morbid obesity) (6, 39). Long-term follow-up studies also suggest that reoperation for recurrence can accumulate over time, reaching up to 12% over ten years in some cohorts, highlighting the potential for downstream cost accrual beyond the index admission (40). Nevertheless, because postoperative outcomes and long-term follow-up were not comprehensively captured in this cost-focused analysis, these data cannot support comparative conclusions regarding surgical strategy or overall value.

## Limitations

Despite its strengths, our study has several limitations. First, the retrospective, single-center design may limit the generalizability of our findings to other populations and healthcare settings. Second, the discharge-record data lacked several relevant variables, including detailed socioeconomic information, patient-reported outcomes, and indirect costs. In addition, cost components were available only as aggregated billing categories; thus, we could not further disaggregate material cost into item-level consumables (e.g., specific meshes, fixation devices, or laparoscopic disposables) to quantify their individual contributions or directly determine the drivers of observed temporal changes. Given the long study period, costs were not adjusted for inflation or healthcare price reforms; therefore, temporal trends may reflect both changes in utilization and changes in unit prices and policy reforms. Third, key clinical and perioperative variables, such as hernia characteristics, body mass index, American Society of Anesthesiologists physical status, and standardized postoperative complication data, were unavailable, which may have resulted in residual confounding. Accordingly, variables such as length of stay and ICU admission should be interpreted as downstream markers of care intensity rather than fully independent effects. Finally, some sociodemographic variables, particularly marital status, were included as proxy indicators of social support and healthcare utilization, but their direct clinical relevance to umbilical hernia is limited, and the marked imbalance across categories (e.g., marital status, patient source, and ethnicity) may have reduced interpretability. Recurrence was rare and identified only from inpatient records within the same institution, so recurrence-related findings should be considered descriptive and interpreted cautiously. More clinically relevant variables, such as parity or pregnancy history, were also unavailable. Future prospective, multi-center studies incorporating these clinical severity and complication measures are warranted to confirm and extend our findings.

## Conclusion

Our study provides a comprehensive analysis of factors influencing hospitalization costs in umbilical hernia repair surgery, identifying laparoscopic surgery, mesh utilization, ICU admission, prolonged hospitalization, later admission year, advanced anesthesia techniques, complicated diagnoses, and additional comorbidities as critical determinants. These findings emphasize the necessity of cost-conscious decision-making in clinical practice, effective resource allocation strategies, and healthcare policy interventions aimed at improving surgical efficiency, reducing economic burdens on patients, and promoting equitable healthcare access.

## Data Availability

The data utilized in this study are sourced from Beijing Chao-Yang Hospital, Capital Medical University. However, access to these data is subject to institutional restrictions, as they were obtained under license for research purposes and are not publicly accessible. Researchers interested in accessing the data may request it from the author (Xiaoli Liu, xiaolil916@163.com), provided that appropriate permissions are obtained from Beijing Chao-Yang Hospital, Capital Medical University.
