# Supplementary material for: Trends and determinants of hospitalization costs for umbilical hernia: a 13-year retrospective analysis from 2012 to 2024
Source: Front Surg. 2026 Apr 9;13:1748565. doi: 10.3389/fsurg.2026.1748565 (PMC13102830; doi:10.3389/fsurg.2026.1748565)
Supplement: Supplementary file 1 [file Supplementaryfile1.docx]

**Supplementary Table S1. Multicollinearity diagnostics for covariates included in the multivariable model**

| **Variable** | **Df** | **VIF** |
| --- | --- | --- |
| Gender | 1 | 1.14 |
| Age group | 3 | 1.09 |
| Admission year group | 2 | 1.12 |
| Patient source | 1 | 1.13 |
| Marital status | 1 | 1.04 |
| Ethnicity | 1 | 1.01 |
| Number of hospitalizations | 2 | 1.02 |
| Payment method | 1 | 1.13 |
| Admission route | 1 | 1.80 |
| Length of hospital stay (days) | 3 | 1.07 |
| Primary diagnosis | 3 | 1.24 |
| Number of additional diagnoses | 3 | 1.06 |
| Surgery type | 1 | 1.44 |
| Mesh use | 1 | 1.12 |
| Primary surgery grade | 2 | 1.16 |
| Primary anesthesia method | 2 | 1.17 |
| Intensive care unit | 1 | 1.63 |
| Ventilator use | 1 | 1.53 |

**Supplementary Table S2. Interaction analyses of temporal changes in cost drivers for umbilical hernia hospitalization**

| **Interaction / Predictor** | **Period** | **Contrast** | **F statistic** | **% change in cost** | **95% CI** | **P value** |
| --- | --- | --- | --- | --- | --- | --- |
| **Admission year × Surgery type** | — | — | 4.525 | — | — | 0.011 |
| **Admission year × Mesh use** | — | — | 17.909 | — | — | <0.001 |
| Surgery type | 2012–2015 | Laparoscopic vs Open | — | 68.1 | (44.3 ~ 95.8) | <0.001 |
| Surgery type | 2016–2019 | Laparoscopic vs Open | — | 112.7 | (94 ~ 133.2) | <0.001 |
| Surgery type | 2020–2024 | Laparoscopic vs Open | — | 110.7 | (96 ~ 126.5) | <0.001 |
| Mesh use | 2012–2015 | Yes vs No | — | 21.0 | (-7.4 ~ 58.2) | 0.162 |
| Mesh use | 2016–2019 | Yes vs No | — | 71.4 | (48.7 ~ 97.6) | <0.001 |
| Mesh use | 2020–2024 | Yes vs No | — | 150.1 | (125.4 ~ 177.4) | <0.001 |

**Notes:** Interaction analyses were performed using multivariable log-linear regression models, with the natural logarithm of total hospitalization cost as the dependent variable. All models were adjusted for the same prespecified covariates as those included in Table 4. Overall interaction effects between admission period and key surgical factors were evaluated using nested model comparisons based on F tests. Period-specific marginal effects were estimated from the interaction models. Percentage change in cost was calculated as (exp(β)−1)×100. Confidence intervals were back-transformed accordingly. Abbreviations: CI, confidence interval.

**Supplementary Table S3. Multivariable analysis of factors associated with total hospitalization cost among patients undergoing open umbilical hernia repair (stratified by surgical approach)**

| **Variable** | **Group** | **β** | **Standard error** | **% change in cost** | **95% CI (% change)** | **P value** |
| --- | --- | --- | --- | --- | --- | --- |
| Sex | Male | Ref. |  |  |  |  |
|  | Female | -0.018 | 0.041 | -1.8 | (-9.4 ~ 6.4) | 0.652 |
| Age group (years) | 18–41 | Ref. |  |  |  |  |
|  | 42–58 | -0.007 | 0.062 | -0.7 | (-12.2 ~ 12.2) | 0.907 |
|  | 59–70 | -0.052 | 0.064 | -5 | (-16.3 ~ 7.7) | 0.421 |
|  | 71–96 | -0.061 | 0.066 | -5.9 | (-17.3 ~ 7) | 0.353 |
| Admission year | 2012–2015 | Ref. |  |  |  |  |
|  | 2016–2019 | 0.125 | 0.075 | 13.3 | (-2.2 ~ 31.2) | 0.096 |
|  | 2020–2024 | 0.249 | 0.07 | 28.3 | (11.9 ~ 47.2) | <0.001 |
| Patient source | Beijing | Ref. |  |  |  |  |
|  | Other provinces | 0.021 | 0.05 | 2.1 | (-7.4 ~ 12.6) | 0.679 |
| Marital status | Unmarried | Ref. |  |  |  |  |
|  | Married | 0.012 | 0.075 | 1.2 | (-12.7 ~ 17.4) | 0.873 |
| Ethnicity | Han | Ref. |  |  |  |  |
|  | Minority | 0.007 | 0.083 | 0.7 | (-14.5 ~ 18.5) | 0.935 |
| Number of hospitalizations | 1 time | Ref. |  |  |  |  |
|  | 2 times | -0.093 | 0.057 | -8.9 | (-18.5 ~ 1.8) | 0.099 |
|  | ≥3 times | -0.017 | 0.078 | -1.7 | (-15.6 ~ 14.6) | 0.830 |
| Payment method | Non-medical insurance | Ref. |  |  |  |  |
|  | Medical insurance | 0.062 | 0.06 | 6.4 | (-5.5 ~ 19.7) | 0.307 |
| Admission route | Emergency | Ref. |  |  |  |  |
|  | Outpatient | -0.009 | 0.087 | -0.9 | (-16.5 ~ 17.6) | 0.916 |
| Length of hospital stay (days) | 1–5 | Ref. |  |  |  |  |
|  | 6–7 | 0.371 | 0.053 | 44.9 | (30.4 ~ 60.9) | <0.001 |
|  | 8–9 | 0.560 | 0.058 | 75.1 | (56.1 ~ 96.4) | <0.001 |
|  | 10–42 | 0.784 | 0.048 | 118.9 | (99.4 ~ 140.4) | <0.001 |
| Primary diagnosis | Umbilical hernia | Ref. |  |  |  |  |
|  | Incarcerated umbilical hernia | 0.003 | 0.097 | 0.3 | (-17 ~ 21.3) | 0.974 |
|  | Umbilical hernia with obstruction | 0.144 | 0.097 | 15.5 | (-4.5 ~ 39.7) | 0.136 |
|  | Gangrenous umbilical hernia | 0.419 | 0.315 | 52 | (-18.2 ~ 182.5) | 0.185 |
| Number of additional diagnoses | 0 | Ref. |  |  |  |  |
|  | 1 | 0.043 | 0.095 | 4.4 | (-13.4 ~ 25.8) | 0.654 |
|  | 2 | 0.122 | 0.087 | 13.0 | (-4.7 ~ 33.9) | 0.159 |
|  | ≥3 | 0.287 | 0.076 | 33.2 | (14.8 ~ 54.7) | <0.001 |
| Primary surgery grade | Grade 2 | Ref. |  |  |  |  |
|  | Grade 3 | 0.054 | 0.054 | 5.6 | (-5 ~ 17.3) | 0.314 |
|  | Grade 4 | 0.173 | 0.106 | 18.9 | (-3.5 ~ 46.5) | 0.104 |
| Primary anesthesia method | Local anesthesia | Ref. |  |  |  |  |
|  | General anesthesia | 0.216 | 0.039 | 24.1 | (14.9 ~ 34.1) | <0.001 |
|  | Combined anesthesia | 0.243 | 0.067 | 27.5 | (11.8 ~ 45.5) | <0.001 |
| ICU | No | Ref. |  |  |  |  |
|  | Yes | 0.600 | 0.106 | 82.2 | (47.8 ~ 124.6) | <0.001 |
| Ventilator use | No | Ref. |  |  |  |  |
|  | Yes | 0.154 | 0.157 | 16.6 | (-14.3 ~ 58.7) | 0.327 |
| Mesh use | No | Ref. |  |  |  |  |
|  | Yes | 0.410 | 0.063 | 50.7 | (33.2 ~ 70.5) | <0.001 |

**Model note:** Log-linear multivariable regression; results are presented as β coefficients with corresponding **percent change in cost** ((exp(β)−1)×100).
**Reference categories:** As shown (Ref.).
**Abbreviations:** ICU, intensive care unit.

**Supplementary Table S4. Multivariable analysis of factors associated with total hospitalization cost among patients undergoing laparoscopic umbilical hernia repair (stratified by surgical approach)**

| **Variable** | **Group** | **β** | **Standard error** | **% change in cost** | **95% CI (% change)** | **P value** |
| --- | --- | --- | --- | --- | --- | --- |
| Sex | Male | Ref. |  |  |  |  |
|  | Female | 0.016 | 0.029 | 1.6 | (-4.0 ~ 7.5) | 0.581 |
| Age group (years) | 18–41 | Ref. |  |  |  |  |
|  | 42–58 | 0.029 | 0.034 | 3.0 | (-3.7 ~ 10.2) | 0.393 |
|  | 59–70 | 0.003 | 0.039 | 0.3 | (-7.1 ~ 8.4) | 0.934 |
|  | 71–96 | 0.045 | 0.044 | 4.6 | (-4.1 ~ 14.1) | 0.309 |
| Admission year | 2012–2015 | Ref. |  |  |  |  |
|  | 2016–2019 | 0.317 | 0.045 | 37.2 | (25.8 ~ 49.8) | <0.001 |
|  | 2020–2024 | 0.393 | 0.047 | 48.1 | (35.2 ~ 62.3) | <0.001 |
| Patient source | Beijing | Ref. |  |  |  |  |
|  | Other provinces | -0.016 | 0.036 | -1.6 | (-8.3 ~ 5.5) | 0.645 |
| Marital status | Unmarried | Ref. |  |  |  |  |
|  | Married | -0.121 | 0.068 | -11.4 | (-22.5 ~ 1.2) | 0.074 |
| Ethnicity | Han | Ref. |  |  |  |  |
|  | Minority | 0.072 | 0.056 | 7.5 | (-3.6 ~ 20.0) | 0.195 |
| Number of hospitalizations | 1 time | Ref. |  |  |  |  |
|  | 2 times | -0.010 | 0.058 | -1.0 | (-11.6 ~ 10.9) | 0.864 |
|  | ≥3 times | 0.007 | 0.103 | 0.7 | (-17.7 ~ 23.2) | 0.948 |
| Payment method | Non-medical insurance | Ref. |  |  |  |  |
|  | Medical insurance | -0.096 | 0.043 | -9.1 | (-16.5 ~ -1.0) | 0.028 |
| Admission route | Emergency | Ref. |  |  |  |  |
|  | Outpatient | -0.008 | 0.094 | -0.8 | (-17.4 ~ 19.3) | 0.935 |
| Length of hospital stay (days) | 1–5 | Ref. |  |  |  |  |
|  | 6–7 | 0.388 | 0.037 | 47.4 | (37.2 ~ 58.3) | <0.001 |
|  | 8–9 | 0.406 | 0.041 | 50.2 | (38.7 ~ 62.6) | <0.001 |
|  | 10–42 | 0.456 | 0.043 | 57.7 | (45.0 ~ 71.5) | <0.001 |
| Primary diagnosis | Umbilical hernia | Ref. |  |  |  |  |
|  | Incarcerated umbilical hernia | -0.196 | 0.117 | -17.8 | (-34.6 ~ 3.4) | 0.094 |
|  | Umbilical hernia with obstruction | 0.020 | 0.133 | 2.0 | (-21.4 ~ 32.4) | 0.882 |
| Number of additional diagnoses | 0 | Ref. |  |  |  |  |
|  | 1 | 0.122 | 0.054 | 13.0 | (1.7 ~ 25.5) | 0.023 |
|  | 2 | 0.206 | 0.052 | 22.9 | (10.9 ~ 36.2) | <0.001 |
|  | ≥3 | 0.287 | 0.048 | 33.2 | (21.3 ~ 46.3) | <0.001 |
| Primary surgery grade | Grade 2 | Ref. |  |  |  |  |
|  | Grade 3 | 0.154 | 0.146 | 16.6 | (-12.4 ~ 55.2) | 0.293 |
|  | Grade 4 | 0.183 | 0.148 | 20.1 | (-10.2 ~ 60.6) | 0.217 |
| Primary anesthesia method | Local anesthesia | Ref. |  |  |  |  |
|  | General anesthesia | 1.049 | 0.169 | 185.4 | (104.8 ~ 297.9) | <0.001 |
| ICU | No | Ref. |  |  |  |  |
|  | Yes | 0.248 | 0.169 | 28.2 | (-7.9 ~ 78.4) | 0.141 |
| Ventilator use | No | Ref. |  |  |  |  |
|  | Yes | 0.075 | 0.191 | 7.8 | (-25.9 ~ 56.8) | 0.695 |
| Mesh use | No | Ref. |  |  |  |  |
|  | Yes | 1.031 | 0.055 | 180.3 | (151.8 ~ 212.1) | <0.001 |

**Model note:** Log-linear multivariable regression; results are presented as β coefficients with corresponding **percent change in cost** ((exp(β)−1)×100).
**Reference categories:** As shown (Ref.).
**Abbreviations:** ICU, intensive care unit.
